# Characteristics of a Successful Nurse Peer Champion in the Implementation of Innovative Digital Technologies in Hospitals: A Qualitative Study

**DOI:** 10.1016/j.pecinn.2024.100339

**Published:** 2024-08-31

**Authors:** Olga Sophia Siebeck, Ciska Hoving

**Affiliations:** Department of Health Promotion, Care and Public Health Research Institute, Maastricht University, P.O. Box 616, 6200 MD Maastricht, the Netherlands

**Keywords:** Implementation, Role models, Healthcare professionals, Nurses, Peer champions, Digital transformation, E-health

## Abstract

**Objectives:**

Using the Motivational Theory of Role Modelling as a framework, this study explores which attributes nurses deem essential for an effective peer champion, particularly in digital transformation processes within hospitals.

**Methods:**

A qualitative study was conducted with semi-structured interviews. Transcripts were coded using a hybrid approach of inductive and deductive coding and analysed using thematic analysis.

**Results:**

Ten nurses from Germany participated. The attributes most often mentioned were competence, taking on responsibility, a positive and passionate attitude, transferring knowledge and supporting aspirants in applying it, and leadership skills. Four types of champions were identified: a pragmatic and structured champion, a passionate innovator, a social and outgoing team leader, and a calm and empathetic team leader.

**Conclusions:**

The findings largely align with the body of literature on peer champion characteristics in other populations and should therefore be used to guide peer champion application in hospitals to enhance effective implementation of innovations.

**Innovation:**

The identification of four unique champion types offers an innovative contribution to the field. Highlighting the unique requirements of nurses when implementing innovative technologies in healthcare, this study emphasises the importance of involving end-users in the design and implementation process of new technologies, a crucial step towards a more sustainable and user-centred digital health ecosystem.

## Highlights

• Four distinct champion types identified; Deploying a mix of champion types ensures a comprehensive coverage of nurse needs.
• Naturally emerging champions significantly contributed to successful nursing technology implementation.
• Competence, responsibility, positive attitude, knowledge transfer, and passion emerged as effective champion attributes.
• Effective champions influenced not just knowledge but attitudes and team dynamics during technology adoption.
• Guidelines for deploying champion teams of various types can enhance implementation effectiveness.

## 1 Introduction

The complex demands of the healthcare sector, such as an ageing population, workforce shortages, and chronic, complex diseases require efficient, cost-effective, and sustainable care [1]. Innovative technologies and equipment, including digital solutions, are fundamentally transforming healthcare systems and play a pivotal role in enhancing patient safety and providing efficient, cost-effective, and sustainable care [2,3]. Integrating these technologies requires significant adaptions in workflows, roles, and delivery [4]. Successful implementation of new technologies can lead to improved clinical outcomes and patient satisfaction, emphasizing the significance of targeted approaches to technology adoption in healthcare settings [5]. Often times, however, new technologies are implemented without considering user needs, such as healthcare professionals applying those technologies [6].

A significant barrier during implementation is the acceptance of new technology [7], and implementation failure remains a considerable problem: depending on how failure is defined and how much organisational change is involved, failure rates range from 30 % to 90 % [8]. Such high failure rates jeopardise the potential benefit of innovation in medicine, health promotion, and public health for patients and communities [9]. In order for digital innovations to be implemented and sustained, support of the healthcare workforce is essential. Nurses make up the largest group of healthcare workers globally, positioning them as key agents to shape the future of digital health [10,11].

To improve implementation outcomes and sustainability of clinical programs, user-centred designs such as peer champions have been found to be very promising [12,13]: “A very important, and sometimes the most important, antecedent to a successful implementation of a mission critical information system is a ‘champion’ for the new system” [14], p. 355. Peer champions are considered a critical component of successful implementation of new workflows or technologies outside and within healthcare settings [[15], [16], [17], [18]]. They are devoted individuals who “volunteer or are appointed to enthusiastically promote and facilitate implementation of an innovation” [15], p. 2. The Motivational Theory of Role Modelling by Morgenroth et al. [19] provides a theoretical foundation for understanding why peer champions can be effective, by illustrating the psychological and motivational processes of those looking up to role models. This theory supports the idea that individuals choose role models based on their ideals and ambitions, which can enhance the influence of peer champions. As Farthing et al. [20] point out, the theory offers the most comprehensive framework for evaluating the role model function and helps to critically determine the mechanisms involved.

In an integrative review on champions in healthcare-related implementation, nearly all reviewed studies reported positive and significant associations with implementation outcomes; furthermore, champions were identified as a key factor for successful implementation by over 80 % of the studies [18]. Notably, most of the literature focuses on the strategies and resources of effective leaders, while relatively little attention is given to the attributes of individuals who lead implementation efforts [21]. George et al. [16] interviewed clinician champions and colleagues to investigate how and why the colleagues got motivated to adopt a new intervention and found that champions who were described as intrinsically motivated, persistent, optimistic, considerate, high-performing, and empathetic, were perceived as effective**.** Bonawitz et al. [18] identified six key attributes for effective champions: influence, intrinsic motivation to take responsibility for the success of the implementation, physical presence, grit, communication skills, enthusiasm and passion, and a participative leadership style.

While involving them to varying degrees, none of the studies have exclusively focused on what nurses perceive as important in an implementation champion. This study addresses this gap by conducting interviews with nurses from various German hospitals with the aim to explore their preferences and needs for and from an effective peer champion, thereby contributing to the limited literature on nurse peer champions within the context of implementation of innovative technologies in hospitals.

To address how others perceive peer champions, the Motivational Theory of Role Modelling (19) will be used as the theoretical foundation. The perception of three essential role model qualities is proposed as key to the influence of role models: goal embodiment, attainability, and desirability, and those who look up to the role models are referred to as “role aspirants” [19].

Focusing on nurse role aspirants' views is expected to expand on the findings of George et al. [16], Miech et al. [18], and Bonawitz et al. [21] by the shedding light onto the attributes nurses perceive as necessary in an influential champion, and how they select a champion among themselves. The findings are expected to contribute to an effective utilisation of peer champions among nurses, thereby improving implementation outcomes in hospital settings.

## 2 Methods

### 2.1 Study design

Given the explorative nature of the research questions, a qualitative study design was chosen. Hamilton et al. [22] emphasize that qualitative research methods are a fundamental part of implementation research, as they allow for explaining organisational change processes, investigating interpersonal dynamics, and identifying facilitators and barriers to intervention adoption. They are also increasingly recognized for their utility in clinical and health research [22]. A qualitative approach allows for a deeper exploration of individual perceptions around peer champions than a quantitative approach.

The research proposal was approved by the ethics assessors of the master's Health Education and Promotion programme at Maastricht University, and the ethical approval was registered under the number FHML/HEP_2023.633. Furthermore, the workers' council and the data protection officer of the Robert-Bosch Hospital in Stuttgart approved of the research proposal, the informed consent form, and the interview guide. The Consolidated criteria for reporting qualitative research (COREQ), [23] detailed in supplementary material 1.

### 2.2 Reflexivity

OS, a native German speaker, conducted and translated 100 % of the interviews herself. OS conducted this research as part of her master's thesis at Maastricht University. OS, a trained nurse with a BSc in European Public Health and an MSc in Health Education and Promotion, previously worked at the hospital where most interviews took place, and two participants were known to her beforehand. She lived in Stuttgart part-time, hence the hospital was selected. This was OS's first systematic experience with conducting a thematic analysis. Being familiar with the workflow of nurses likely influenced OS's approach to the interviews and the accommodations she was able to make for the nurses. Author CH supervised the research and has extensive experience in conducting and supervising qualitative analysis.

### 2.3 Sampling

A combination of criterion, convenience and snowball sampling was performed. These sampling methods are non-probabilistic and can often be implemented more quickly than probabilistic methods, as members of the target population meet practical criteria such as geographical proximity and easy accessibility [24]. To ensure that all participants met the study's criteria, we employed criterion sampling, a method frequently utilized in implementation research [25].

Inclusion criteria were a nursing degree and working experience in a hospital in Germany, and exclusion criteria were working outside hospitals (GP office, nursing homes, elderly homes). The final sample comprised 10 nurses.

Initial recruitment was conducted at Robert-Bosch Hospital in Stuttgart, Germany during an in-person meeting with nursing management, via social media, and on LinkedIn. Interested participants were scheduled for an interview and received an informed consent form detailing the study's content, participant's rights, and confidentiality and data protection. Informed consent was also discussed and signed at the onset of the interviews.

### 2.4 Data collection

Ten expert interviews were conducted between May and June 2023, four in person and six online. The interviews averaged 13.35 min, ranging from six to 25 min. To accommodate nurses' busy schedules, most initially planned 20–30-min interviews were shortened to 10–15 min. The semi-structured interviews featured open-ended questions, which allowed participants to elaborate on their own experiences while providing structure to the interview process. Participants were briefly informed about the purpose of the study and any remaining questions were answered. The interview started with demographic questions about the participants' age, degree, nursing experience, unit, and current position.

The first version of the interview guide was tested in a pilot interview (*n* = 1) and included detailed questions about the three domains of the Motivational Theory of Role Modelling: Goal embodiment, attainability, and desirability, as well as questions about the participants' experience and their views on role models. Feedback from the pilot interview revealed that such detailed questions were infeasible for short interviews as they as they require deep immersion and reflection on the theory. The interview guide was revised for accessibility, and the new version included open questions about the characteristics the participants perceived as important for a good role model, their own experiences as role aspirants, and their expectations for an ideal role model. The questions were open-ended; the introductory question asked the participant to recall an instance where a new technology was implemented in their workplace and identify someone who acted as a role model, followed up by questions about what made these champions effective. While the interviews were short in duration, the respondents offered nuanced and detailed responses, and rich data sharing was encouraged by asking the respondents to elaborate on specific examples. Key themes and patterns, such as the different champion attributes, began to emerge early in the process of data collection and were reinforced consistently over the subsequent interviews, indicating richness and saturation of data.

The interviews were conducted and transcribed in German, with coding done in English. Recordings were transcribed verbatim, then deleted, and the transcripts were imported to Atlas.ti and stored on Surfdrive. Quotes in the paper were translated from German to English using DeepL [26] and edited for clarity. Both versions of the interview guide can be found in supplementary material 2.

### 2.5 Data analysis

A thematic analysis was conducted following the six steps approach by Braun et al. [27]. Coding used a hybrid approach, combining “the two contrasting philosophical methods of reasoning: “a top-down, deductive, theoretical process and a bottom-up, inductive, data-driven process” [28] p. 7). It combines a set of a priori developed codes based on theory or existing data with codes that are a posteriori derived from the interview data. It was deemed suitable for this study, as the aim was to explore participants' experience with peer champions against the background of the theory. This approach was suitable for exploring participants' experience with peer champions against the background of the Motivational Theory of Role Modelling, A preliminary set of codes was derived from the theory and applied to the first interviews. If attributes in the data did not fit the prespecified codes, new codes were added inductively. The codes were combined to form a coding tree, which can be found in supplementary material 3 and comprised 43 codes in total.

In qualitative research, it is standard to assess interrater reliability by coding a subset of the data, often around 10 %. Campbell et al. [29] note that coding a small portion, such as 10 %, is a typical approach for ensuring consistency across coders, which enhances the reliability of the study's findings.

OS and a second researcher tested interrater reliability after the second researcher was introduced to the theory and the codebook. Both researchers independently coded the same transcript (*n* = 1, which equals 10 %), resolved discrepancies and discussed themes, achieving a percentage agreement of 83 % and a Cohens' Kappa of 0.77, indicating substantial agreement [30,31]. Interrater reliability coding was conducted using Microsoft Word and Atlas.ti (Version 23.1.0), and afterwards exclusively with Atlas.ti. As both researchers are native German speakers fluent in English, the German interviews were coded with English coding tree by both researchers and compared for coherence. One researcher coded the remaining interviews during several rounds of coding, adding new codes iteratively as necessary.

The codes were categorised into themes according to the theory: acting as a behavioural model, representing the possible, and being an inspiration. Interview data codes were first sorted by to frequency mentioned across interviews, then mapped to show their connections, co-occurrences and associations (supplementary material 4). Theory-based codes, coloured dark green, white, and brown in the map, were excluded from further analysis, as it emerged that this would go beyond the scope of this study.

## 3 Results

### 3.1 Participant characteristics

Of the 10 participants, five were women, and five were men. The mean age was 39.7 (range 25–63), and the mean years of clinical experience were 13.7 years (range 1–42). All participants held a nursing degree.^2^ The majority worked in direct patient care at the time of the interview, two had previously changed to other fields, and almost half were currently pursuing an advanced degree. For detailed information, see Table 1.

**Table 1:** Descriptive characteristics of participants (*n* = 10).

Variable	
Age (mean, range)	39.7 (25–63)
Female gender	50 %

Highest level of education	
*Nursing Degree (*i.e.*, Ausbildung)*^*1*^	60 %
*Bachelor's degree*	30 %
*Ph. D*	10 %
Currently enrolled in advanced degree	40 %
Years of clinical experience (mean)	13.7 (1–42)
Head of unit	20 %

Area of clinical experience (n)	
*ICU, ER*	4
*Oncology*	2
*Neurology*	1
*Geriatrics*	1
*Nursing Education*	1
*Quality Management*	1

### 3.2 Analysis in the interviews; Peer champion attributes

Participants described three champions that were heads of their unit, one that held a mentor role, seven that did not have any assigned position, and three that were outside experts from other departments, such as IT. The role model attributes most frequently mentioned across participants were “Competence” (30), “Taking on responsibility” (21), “Positive Attitude” (16), “Transfer of Knowledge” (15), “Doing it yourself” (13), “Team Leadership”/”Team Membership” (12), being “Supportive” (12), and “Passion” (10), which are described in detail and illustrated with interview quotes in the following section.

#### 3.2.1 Competence

All participants highlighted the critical importance of competent champion as performing well in their job, possessing specialist knowledge and knowing how to apply it; being a “computer freak”, and very advanced with new technology. Examples are a colleague from the IT department who was very supportive and helpful through his knowledge, his experience, and his point of view, or team members who attended training for specific technology.

“He was just a (…) computer freak; he knew his way around it very well, he was just a bit of a role model for me, he explained everything to me, he tried things out straight away, he was actually already further ahead than we were trained in school.”

(Participant 7)

#### 3.2.2 Taking on responsibility

The majority of participants expressed a strong appreciation for a champion who takes on responsibility within and above their designated responsibilities as a nurse. Participants described how a champion stepped up and took responsibility for understanding new processes like a new device or software – particularly within the transition from paper-based patient records to a digitally based system. Moreover, a champion who also took responsibility for teaching others and sharing their newly acquired knowledge and skills was highly valued. The additional effort and commitment were not expected but appreciated and perceived as valuable for the implementation process.

“(she) faced the responsibility, did that and yes, actually encouraged us to do that. To go ahead and say ok, someone has to have the courage, and ideally the person who feels the safest on that day will do it. She was not more qualified than the rest, she was a normal nurse with not too many years of experience, she did it, she went ahead, that was impressive. That was someone in the team who then said come on, we have to manage it like that now.”

(Participant 6)

“And he actually did it quite well, he sat down once again and then somehow, I think, he also took things from the Internet (well, what do you mean from the Internet); he always wrote it all down, and he had, so to speak, developed a guideline for us, so to speak, that was actually quite helpful.”

(Participant 10)

#### 3.2.3 Positive attitude

Thirdly, half of the participants described the importance of a champion with a positive attitude when faced with challenges with new technologies, who lifted others up when things seemed hard and maintained a friendly approach.

“And then he also gets upset about it, but he doesn't fall into such a ‘I'm not doing anything anymore’ situation, as many older people do, (…) But he just doesn't fall into such a situation, and I think that's just nice.”

(Participant 9)

#### 3.2.4 Transfer of knowledge

Over half of the participants strongly appreciated peer champions who shared newly acquired skills and knowledge in a structured way that the aspirants could easily follow. While the champions did not have a formal role as a trainer or a teacher, sharing knowledge about the new technology and teaching skills, mainly in informal situations such as a one-minute-training on the ward, older and experienced colleagues sharing knowledge they had accumulated over decades, or a champion who simply took the time to explain a new technology very structured and calmly was regarded as highly positive.

“(…) and she knew some cool key combinations where you can, for example, somehow move the day forward in the patient file and so on. I don't know, she seems extremely organised to me, maybe she's even an equipment officer, or I don't know exactly anymore, but in any case, she's extremely organised and structured. I found her very pleasant at that moment.”

(Participant 1)

“Of course, there were also colleagues from medical technology who had done a lot there and were also always approachable and also colleagues who were at special training programs (…)”

(Participant 8)

#### 3.2.5 Doing it yourself

In addition to sharing knowledge and skills, half of the participants highly valued champions who motivated role aspirants to put the new skills into practice soon as possible, so they would maintain motivation to master it. Furthermore, participants valued an opportunity to contribute their own thoughts and ideas to the implementation process and appreciated role models that made them feel acknowledged and included.

“So I just found that fascinating, how he dealt with it and I just looked at that a bit more, because usually it's better to just try it, as if you just resist it.”

(Participant 7)

“To stand up for the topic, to say hey, this is the guideline, we have to implement it and then to clarify that and then also to let the team participate, so to speak. So everyone had the chance to express things, for example, where something could still be changed or what ideas they had.”

(Participant 7)

#### 3.2.6 Team Leadership/Team Membership

The champion's ability to work as a team and lead others was highly valued by the participants, expressed as having a good standing in and being accepted by the team, regardless of whether they were an actual member of the team or an external colleague.

“So I think that is totally important that the person has a good standing in the team, that she is a part of the team, so I have experienced that when someone comes from the outside and says here is how you do it now - as an employee you automatically go into such an anti-attitude and say ‘okay, you don't know our processes at all, how are you going to tell me how I should do it all at once?’”

(Participant 3)

#### 3.2.7 Supportive

Several participants appreciated a supportive peer champion who alleviated anxieties and worries associated with using new technology by reassuring them not to worry too much, repeatedly going through the process, or being available to address any problems that occurred.

“(…) and they are afraid that(…) during the night shift when you are alone and you don't know how to use the equipment, he always says, “Look at it, you can do it, you have expertise - so he always encouraged you in the sense of what we are doing. And I think that helped me a lot.”

(Participant 5)

“He has always dealt with the technology and he has never left us alone, so it was always a very positive image and whenever I was overwhelmed, the situation was like: Come on, we'll take a look at it (now), it's not that bad, it doesn't hurt, nothing can happen and I'll show you, and it was more like this support, (…) I always had the feeling that I can count on this person in everyday life, even if it was so stressful on the ward, the ward manager was still always there, always approachable, and above all, he already had the specialist knowledge about all the equipment, so ultimately it was like a mentor principle.”

(Participant 5)

#### 3.2.8 Passion

Similar to a positive attitude, passion was considered a major requirement by half of the participants: Working on their own, going the extra mile and pushing boundaries; caring about their own and others' progress, all stemming from intrinsic motivation: People who “(…) radiate that it's cool to try something new” as something that helped them engage with unfamiliar tasks.

“That was a ward that we opened ourselves, so to speak, and it was about somehow bringing in a bit of new things and trying out new things, what you can change or do new in nursing, especially with digitalisation, which is always a bit difficult at the university hospital, because there are always quite a few things that you have to adhere to, and he also always tried to somehow push things forward and introduce new things and took care of it a lot, so he was just a very driving force and just also very nice and friendly and just always got along well with everyone, somehow.”

(Participant 10)

“I think it's also important what kind of character the person has, that you're contagious. So, there are people who can inspire you with new things, who have such an aura, and who somehow radiate that it's cool to try something new. Yes, I think professional experience, character, a good standing within the team, being part of the team.”

(Participant 3)

## 4 Discussion and conclusion

### 4.1 Discussion

This study aimed to investigate the characteristics nurse role aspirants perceive as valuable in a peer champion and how they identify a peer champion among them. Participants mainly described effective champions as competent, supportive, willing to take on responsibility without being distinctly asked to do so; sharing specialised knowledge and skills and encouraging others to put the skills into practice, positive and passionate, and being a team member and skilled leader.

Competence being the characteristic most frequently mentioned is in alignment with champion influence tactics found in a 2015 review [32], where tech-savvy champions spent considerable amounts of time on redesigning workflows and mitigating technical difficulties. Bonawitz et al. (2020) observed characteristics similar to what this study calls “taking on responsibility” in their study which they defined as “personal responsibility for the implementation of the initiative's success” [21]. Bonawitz et al. (2020) who found that effective champions “exuded a ‘contagious passion’ that helped colleagues understand (…)” [21], p. 7; and Miech et al. (2018), who found that effective champions maintain a positive focus and drive the implementation with energy and enthusiasm [18]. These characteristics resemble champions who push boundaries and drive innovation in their environments; also defined as “innovator” by Rogers [33], which inspired the champion type name and indicates the substantial importance of a positive and passionate attitude in champion. When confronted with unfamiliar tasks or tools, a supportive champion was deemed important. A champion that anyone could approach – young, digitally versed nurses just as much as older ones who did not know anything about computers. Here, the champion also took on a mentoring role. Using instructional techniques that encouraged “learning by doing” seemed to be an important element, which links back to the behavioural domain of the Motivational Theory of Role Modelling: “Vicarious Learning” as a mechanism and “Skill Acquisition”. This highlights the critical importance of a present, hands-on champion who shows aspirants how to put the newly acquired skill directly into practice, supports the aspirants who encounter challenges with new technology, and is receptive and available.

Contrary to what was expected, conscious motivational processes regarding aspirants' goals and the champion selection process did not emerge from the interviews as a topic the participants were concerned with. In the majority of cases, the champion either took on the role voluntarily, without being asked to do so, or they held a formal position which predisposed them to act as a champion.

#### 4.1.1 Peer champion types

Reoccurring themes in the data were a high level of competence, taking on responsibility without being distinctly asked to do so; to share specialised knowledge and skills and encouraging others to put the skills into practice, positive and passionate, and being a team member and skilled leader. Using the previously created attribute map, attributes that describe similar champion qualities were grouped together and colour-coded in an additional step of analysis (such as “passion”, “positive attitude”, and “pushing boundaries” or “calm attitude”, “reliability”, and “safety”). This process resulted in four clusters of attributes provided the foundation for the following four types of champions: Structured Pragmatist, Innovator, Social Team Leader, Calm Team Leader (see Table 2).

**Table 2:** Champion types.

Name	Codes
*Structured Pragmatist*	Keeping it Simple, Experience, Practicality, Pragmatism, Professionalism, Structured Approach
*Innovator*	Honesty, Passion, Positive Attitude, Pushing Boundaries, Setting Boundaries, Taking on Responsibility
*Social Team Leader*	Communication Skills, Honesty, Setting Boundaries, Social Skills, Supporting, Team Leadership, Team Member (Shared Group Membership), Trustworthiness
*Calm Team Leader*	Calm Attitude, Empathy, Eye Level, Reliability, Safety, Supporting, Trustworthiness, Reliability, Encouraging, Doing it yourself

Although there was considerable overlap, each participant's definition of an effective peer champion was unique. This suggests that there is not a singular, ideal peer champion archetype. Instead, depending on their individual circumstances, different types of champions might prove beneficial to aspirants. In order to deploy the best champion for a given demographic or occupational group, investigating the efficacy of particular types in various contexts may provide valuable insights. This is of considerable importance, as rapid changes in work environments, such as transforming paper-based into digitally based workflows, can lead to uncertainties, stress, and increased pressure on employees [34], which an effective champion could mitigate. Several participants mentioned that older nurses struggle with adapting to using digital technologies in their routines, and to them, a champion with the right characteristics could be of significant value. Moreover, considering that the nursing sector in Germany is just as affected by technological, specifically digital transformation as other jobs, champions adept in technology and empathy play a crucial role in building widespread support and effectively guiding stakeholders through implementation processes.

Considering the different attributes nurses preferred and the different champion types that help them, learning theories focused on social processes might aid in explaining these findings. Social Learning Theory by Albert Bandura proposes that people learn through observing others following the three steps observation, imitation, and modelling [35]. Connectivism as proposed by Siemens and Downes refers to learning as a process that connects information sources and networks and was constructed to complement the previous learning theories of Behaviourism, Cognitivism, and Constructivism [36]. Connectivism acknowledges that learning processes have changed with the evolution of technology and that management and leadership need to contain different viewpoints for exploring ideas completely [36].

Future research should focus on the following four avenues. Firstly, as most participants worked at “innovative” institutions and were higher educated than the average nurse, it can be assumed that they were relatively open to innovation compared to many of their peers. Drawing on the homophily principle as stated by Rogers [28], it is possible that innovative nurses prefer innovative champions, but less innovative nurses prefer someone more like them, i.e., a champion that understands the innovation and leads by example but is closer to aspirants' age or level of education. As the sample was quite homogenous in education and presumed openness to innovation, future research should include nurses with only a vocational nursing degree. Secondly, this study investigated the attributes that make a champion effective, but not which ones do not make a champion effective. To get a comprehensive image of effective champions, investigating the attributes that could be detrimental to champion success might contribute valuable insights. Thirdly, the different champion types were a novel finding in this study and warrant further investigation. A large-scale survey, for instance, which investigates whether nurse have preferences for a certain champion type is necessary to evaluate the significance of the findings of this study. Vignette studies combine traditional survey methodology and a vignette experiment, which allows for a more causal investigation of participant judgements. A vignette study with the different champion types could provide deeper insights. Learning theories might serve as a valuable theoretical foundation.

Lastly, when champions are deployed in implementation processes, their effect on implementation processes should be evaluated, as personal preferences of nurses do not necessarily have to reflect the actual, measurable impact on implementation outcomes.

### 4.2 Innovation

The focus on a tailored approach to understanding the unique challenges of implementing digital technologies in hospitals puts the study in a specific frame of innovation. So far, research on digital transformation in healthcare settings has mostly been centred around technology, focusing on technical, structural, or process-related factors. This study is focused on the perceptions and preferences nurses have for effective peer champions, which offers a novel perspective on implementation processes of innovative technologies, such as digital health technologies in hospitals.

This study identified unique characterisations of what nurses perceive as effective champions, which suggests that there is not a one-size-fits-all strategy for becoming or selecting a peer champion.

The idea that effective championing depends on the context and should be customised to cultural norms, values, and individual requirements of the nursing workforce presents a novel concept to the field of healthcare technology implementation. Finally, by highlighting the specific challenges and unique requirements of nurses when implementing innovative technologies in healthcare, this study paves the way for future investigations into more personalised and effective approaches to digital transformation in healthcare, but also to other technology and equipment such as robots or mobilisation devices for nursing. It emphasises the importance of involving end-users in the design and implementation process of new technologies, a crucial step towards a more sustainable and effective digital health ecosystem.

### 4.3 Strengths and limitations

This study offers in-depth insights into the characteristics nurses in Germany assign to effective peer champions. The adapted interview guide facilitated an open, deep exploration of participants' experiences, opinions, and imaginations, thereby yielding insights beyond the theory. The initially planned interview duration of 20–30 min was reduced to around ten minutes to encourage participation because it became apparent that nurses would not be able to participate in a 30-min interview during working hours. The shorter interviews increased participation and facilitated interviews during participants' shifts. While our sample (*n* = 10) remained rather small, it exhibited a considerable degree of heterogeneity in terms of age and working experience, allowing us to capture a broad range of perspectives.

The current study is also subject to considerable limitations. The participants were recruited by convenience and snowball sampling, which poses a risk of selection bias and low sample variety. Forty percent of participants were currently enrolled in an advanced degree and 20 % were heads of their unit, which is not representative of the average nurse in Germany [37] and might influence their openness to innovation. Furthermore, participants decide to participate in a study based on their needs and personal characteristics, which unintentionally creates self-selection bias [38]. Professionals with higher education are generally more motivated and willing to tackle research issues that emerge from clinical practice and their personal clinical experiences, due to their enhanced understanding of research methodologies, and holding a managerial position in a hospital has been shown to predict higher research participation [39]. Most participants worked in hospitals open to innovation that had already made efforts to facilitate systematic implementation of technology supporting digital transformation. Due to time constraints among the participants and the researcher, the interviews were relatively short. While the answers still were quite heterogeneous, this warrants deeper exploration in a larger study, as this study could be considered a pilot study. Furthermore, it is important to acknowledge a limitation regarding gender representation: 50 % of participants were male, which is a considerable deviation from the usual distribution of male and female nurses in Germany, where in 2022, 83 % of all nurses were female [40]. The generalisability of our findings may be limited by this gender imbalance, particularly concerning perspectives and experiences of female nurses. Conversely, as the share of male nurses is so low, male nurses' views might be underrepresented in the body of literature, therefore this imbalance can also be regarded as a strength of the study.

### 4.4 Conclusion

This study was conducted to investigate what attributes nurses perceive as important in effective peer champions. We identified several important attributes, most of which were in line with existing literature, and clustered them into four types of champions. Participants mainly described emergent champions, opposed to appointed champions. By identifying characteristics of effective champions, this study delivers promising results for targeted utilisation of peer champions in implementation processes. While most of the identified attributes were in line with existing literature, the four champion types are a novel result which can contribute to the effective utilisation of peer champions among nurses and similar occupational groups and has the potential to substantially improve implementation outcomes.

## Funding

This research was conducted as part of the first author's master's programme and did not receive any specific grant form funding agencies in the public, commercial, or not-for-profit sectors.

## CRediT authorship contribution statement

**Olga Sophia Siebeck:** Conceptualization, Methodology, Formal analysis, Investigation, Data curation, Writing – original draft, Writing – review & editing, Visualization, Project administration. **Ciska Hoving:** Conceptualization, Methodology, Writing – review & editing, Supervision, Project administration.

## Declaration of competing interest

The authors declare that they have no known competing financial interests or personal relationships that could influence the work reported in the paper.

The authors confirm all patient/personal identifiers have been removed or disguised so the patient/person(s) described are not identifiable and cannot be identified through the details of the story.

## Appendix A Supplementary data

Completed COREQ checklist Image 1

Interview guide (in German and English) Image 2

Coding tree Image 3

Map of code connections, co-occurences and associations Image 4

## Footnotes

2 In Germany, a nursing degree is not equivalent to a bachelor's degree but obtained through three-year vocational program (Ausbildung). Increasingly more universities offer bachelor's degrees in nursing.
