# Supplementary material for: Characteristics of a Successful Nurse Peer Champion in the Implementation of Innovative Digital Technologies in Hospitals: A Qualitative Study
Source: PEC Innov. 2024 Aug 31;5:100339. doi: 10.1016/j.pecinn.2024.100339 (PMC11460499; doi:10.1016/j.pecinn.2024.100339)
Supplement: Supplementary file 1 — Completed COREQ checklist [file mmc1.pdf]

Consolidated criteria for reporting qualitative studies (COREQ): 32-item checklist

No. Item	Guide questions/description	Reported on Page #
**Domain 1: Research team and reflexivity**		
Personal Characteristics		
1. Inter viewer/facilitator	Which author/s conducted the interview or focus group?	OS
2. Credentials	What were the researcher's credentials? E.g. PhD, MD	BSc, RN
3. Occupation	What was their occupation at the time of the study?	Student
4. Gender	Was the researcher male or female?	female
5. Experience and training	What experience or training did the researcher have?	Bachelor-level research; clinical experience as nurse
Relationship with participants		
6. Relationship established	Was a relationship established prior to study commencement?	yes
7. Participant knowledge of the interviewer	What did the participants know about the researcher? e.g. personal goals, reasons for doing the research	credentials, affiliation to university, reasons for doin research (master's thesis)
8. Interviewer characteristics	What characteristics were reported about the interviewer/facilitator? e.g. Bias, assumptions, reasons and interests in the research topic	Reasons and interests in the research topic

23. Transcripts returned	Were transcripts returned to participants for comment and/or correction?	no
**Domain 3: analysis and findings**		
Data analysis		
24. Number of data coders	How many data coders coded the data?	2 coded 1 transcript, 1 coded remaining 9
25. Description of the coding tree	Did authors provide a description of the coding tree?	yes
26. Derivation of themes	Were themes identified in advance or derived from the data?	a theory was used in advance, and themes were derived from the data
27. Software	What software, if applicable, was used to manage the data?	Atlas.ti
28. Participant checking	Did participants provide feedback on the findings?	no
Reporting		
29. Quotations presented	Were participant quotations presented to illustrate the themes/findings? Was each quotation identified? e.g. participant number	yes
30. Data and findings consistent	Was there consistency between the data presented and the findings?	yes
31. Clarity of major themes	Were major themes clearly presented in the findings?	yes
32. Clarity of minor themes	Is there a description of diverse cases or discussion of minor themes?	minor themes were integrated into major themes

**Domain 2: study design**		
Theoretical framework		
9. Methodological orientation and Theory	What methodological orientation was stated to underpin the study? e.g. grounded theory, discourse analysis, ethnography, phenomenology, content analysis	Thematic analysis
Participant selection		
10. Sampling	How were participants selected? e.g. purposive, convenience, consecutive, snowball	convenience + snowball sampling
11. Method of approach	How were participants approached? e.g. face-to-face, telephone, mail, email	face-to face meetings, flyer in hospitals, email
12. Sample size	How many participants were in the study?	ten
13. Non-participation	How many people refused to participate or dropped out? Reasons?	none
Setting		
14. Setting of data collection	Where was the data collected? e.g. home, clinic, workplace	online via zoom (n=6), in-person at hospital (n=4)
15. Presence of non-participants	Was anyone else present besides the participants and researchers?	no
16. Description of sample	What are the important characteristics of the sample? e.g. demographic data, date	see table 1 in results section
Data collection		
17. Interview guide	Were questions, prompts, guides provided by the authors? Was it pilot tested?	open-ended questions in semi-structured interview. Pilot tested (n=1)
18. Repeat interviews	Were repeat interviews carried out? If yes, how many?	no
19. Audio/visual recording	Did the researcher use audio or visual recording to collect the data?	audio recording
20. Field notes	Were field notes made during and/or after the interview or focus group?	no
21. Duration	What was the duration of the interviews or focus group?	13.35 min on average (6-25)
22. Data saturation	Was data saturation discussed?	yes, but time constraints limited the number of participants
