# Supplementary material for: Characteristics of a Successful Nurse Peer Champion in the Implementation of Innovative Digital Technologies in Hospitals: A Qualitative Study
Source: PEC Innov. 2024 Aug 31;5:100339. doi: 10.1016/j.pecinn.2024.100339 (PMC11460499; doi:10.1016/j.pecinn.2024.100339)
Supplement: Supplementary file 2 — Interview guide (in German and English) [file mmc2.pdf]

Interviewleitfaden Olga Siebeck

Demografische Fragen

- Wie alt sind Sie?
  Was ist Ihr höchster Bildungsabschluss?
- Wie viele Jahre Arbeitserfahrung in der Klinik haben Sie?
- In welchem Bereich arbeiten Sie?
- Was ist die korrekte Bezeichnung Ihrer derzeitigen Position/ Tätigkeit?

Einführungsfrage

- Denken Sie an das letzte Mal (oder an irgendeinen Zeitpunkt in Ihrer Laufbahn), als eine neue Technologie in Ihrer Abteilung eingeführt und umgesetzt wurde. Vielleicht mussten Sie Ihre Routine ändern oder mit einer neuen, anspruchsvollen Technologie arbeiten. Fällt Ihnen ein Kollege (oder eine Kollegin) in Ihrem Team, eine Führungskraft oder sogar jemand aus einer anderen Abteilung ein, der/die Ihnen in dieser Situation als Vorbild diente? Jemanden, den Sie angeschaut haben und bei dem Sie dachten, dass Sie es auch so machen wollen wie er? (

  J/N)

- Erzählen Sie mir von dieser Situation und der Person!
  (je nach Antwort: nachfragen – was genau waren die Verhaltensweisen? Was für Eigenschaften? Warum genau diese Person?)

- stellen Sie sich die ideale Person vor, die (Ihnen) helfen könnte, eine Innovation zu implementieren: Was wäre das für eine Person? **Warum wäre diese Person für Sie vertrauenswürdiger/inspirierender als andere?**
  Weiter nachfragen: Warum diese und jene Verhaltensweisen?

Interview guide Olga Siebeck

Demographic questions

- How old are you?
- What is your highest level of education?
- How many years of clinical work experience do you have?
- In what field do you work?
- What is the correct title of your current position/job?

Introductory question

- Think about the last time (or any time in your career) a new technology was introduced and implemented in your department. Perhaps you had to change your routine or work with a new, challenging technology. Can you think of a colleague (or co-worker) on your team, a manager, or even someone from another department who served as a role model for you in this situation? Someone you looked at and thought you wanted to do it like them?

- (Y/N)

- Tell me about this situation and person!
- (Depending on answer: inquire - what exactly were the behaviors? What kind of characteristics? Why exactly this person?)

- imagine the ideal person who could help (you) implement an innovation: What kind of person would this be? Why would this person be more trustworthy/inspiring to you than others?
  Further inquire: why such and such behaviors?

Appendix 2b: Original interview Questions

The theory outlines the processes that determine the **extent** to which **role models** who **embody role aspirants' goal**s and are **perceived to be desirable and attainable** contributes to the **effectivenes**s of these role models in their three different functions. It discusses how both the attributes of **role models** and of **role aspirants** contribute to these perceptions.

The interview questions are based on the theory in order to answer the research questions, which are

I. From the point of role aspirants, what are the characteristics of a good peer champion?
II. How would role aspirants identify the peer champion among them?
III. To what extent do role aspirants perceive the three characteristics as crucial for champion selection
    a) Acting as a behavioural model (Goal embodiment)
    b) Being inspirational (Desirability)
    C) Representing the possible (Attainability)

Acting as a behavioural model (the **how**)

- When you think of a successful person you admire, what qualities or behaviours do they exhibit that make you believe that you could achieve similar success? Is it important to you that they have achieved a specific goal, or are there any other criteria/ behaviours that you find important?
- Think of a time when a new technology was invented at your workplace, this could be any technology. Can you share/think of an example of a time when you chose a role model (=a colleague) to follow based on their behavior? What behaviors made them trustworthy for you?

Representing the possible (the **what**)

- Can you describe a time when you were motivated to pursue a new goal because of a role model who demonstrated that reaching such a goal was possible? What specific qualities or achievements did this role model have that inspired you? (if not: imagine…)

Being inspirational (the **why**?)

- Can you describe a time when you were inspired to adopt a new goal or aspire toward something different because of a role model (a colleague)? What specific qualities or achievements did this role model have that inspired you? (if not: Image…)
- In your opinion, what are the most important criteria for a role model to be an effective inspiration for you? How does this differ from the criteria you might use to choose a role model who represents the possible or who demonstrates how to achieve a particular goal? (
