# Supplementary material for: Characteristics of a Successful Nurse Peer Champion in the Implementation of Innovative Digital Technologies in Hospitals: A Qualitative Study
Source: PEC Innov. 2024 Aug 31;5:100339. doi: 10.1016/j.pecinn.2024.100339 (PMC11460499; doi:10.1016/j.pecinn.2024.100339)
Supplement: Supplementary file 3 — Coding tree [file mmc3.pdf]

ATLAS.ti Report

Thesis 2023

Codes grouped by Code groups

Report created by Olga Siebeck on 3. Jul 2023

Based on Data

28 Codes:

- ### Calm Attitude

  Created: 25.05.23 by Olga Siebeck, **Modified:** 21.06.23 by Olga Siebeck

  Comment:
  > Champion has a calm attitude, radiates calmness

- ### Communication Skills

  Created: 26.05.23 by Olga Siebeck, **Modified:** 21.06.23 by Olga Siebeck

  Comment:
  > Champion communicates well within the team and with other professions; is able to convey or share ideas and feelings effectively (Oxford Dictionary)

- ### Doing it yourself

  Created: 25.05.23 by Olga Siebeck, **Modified:** 10.06.23 by Olga Siebeck

  Comment:
  > Champion performs certain task themselves, then motivates, enables, or asks role aspirants to do it themselves. Role aspirants are also motivated to bring in their own ideas. Role aspirants perceived this as helpful in learning a new skill.

- ### Empathy

  Created: 14.06.23 by Olga Siebeck, **Modified:** 21.06.23 by Olga Siebeck

  Comment:
  > Peer champion is perceived as being empathetic

- ### Encouraging

  Created: 25.05.23 by Olga Siebeck, **Modified:** 21.06.23 by Olga Siebeck

  Comment:
  > Champion encourages role aspirants to try something new, take on responsibility, be proactive, and be bold.

- ### Experience

  Created: 14.06.23 by Olga Siebeck, **Modified:** 14.06.23 by Olga Siebeck

  Comment:
  > Champion possesses significant, year-long experience in their respective field

- ## Eye Level

Created: 10.06.23 by Olga Siebeck, **Modified:** 21.06.23 by Olga Siebeck

Comment:
> role aspirant has the feeling that the peer champion interacts with him or her on an equal footing (eye level)

- ## Honesty

Created: 09.06.23 by Olga Siebeck, **Modified:** 21.06.23 by Olga Siebeck

Comment:
> Peer Champion is perceived as being honest, even when it might be inconvenient

- ## Keeping it Simple

Created: 28.05.23 by Olga Siebeck, **Modified:** 21.06.23 by Olga Siebeck

Comment:
> Peer Champion provides easily understandable explanations, no unnecessary information

- ## Passion

Created: 08.06.23 by Olga Siebeck, **Modified:** 21.06.23 by Olga Siebeck

Comment:
> Peer Champion is perceived as being passionate about their work or about a specific task they are executing

- ## Perceived Benefit

Created: 10.06.23 by Olga Siebeck, **Modified:** 21.06.23 by Olga Siebeck

Comment:
> Doing a certain taks like the champion gives role aspirant a perceived benefit over how they had done it beforehand

- ## Positive Attitude

Created: 25.05.23 by Olga Siebeck, **Modified:** 21.06.23 by Olga Siebeck

Comment:
> Role Aspirant perceives Peer Champion as having a positive attitude overall and regarding their work

- ## Practicality

Created: 25.05.23 by Olga Siebeck, **Modified:** 10.06.23 by Olga Siebeck

Comment:
> Champion is perceived as being practical, explaining things in a clear way

- ## Pragmatism

Created: 13.06.23 by Olga Siebeck, **Modified:** 13.06.23 by Olga Siebeck

Comment:
> Some sentences were initially coded with “practicality”, but the concept translates better to pragmatism.
> Practicality: Champion has a practical explanation for something they do, stays with the topic
> Pragmatism: Champion approaches a situation in a pragmatic way

- **Professionalism**

  Created: 10.06.23 by Olga Siebeck, **Modified:** 10.06.23 by Olga Siebeck

  Comment:
  > Peer Champion is perceived as being a professional/ acting professionally by colleagues and patients

- **Pushing Boundaries**

  Created: 08.06.23 by Olga Siebeck, **Modified:** 21.06.23 by Olga Siebeck

  Comment:
  > Champion is perceived as pushing boundaries of what is perceived as normal without being asked to do so; intrinsically motivated

- **Reliability**

  Created: 25.05.23 by Olga Siebeck, **Modified:** 21.06.23 by Olga Siebeck

  Comment:
  > Champion is a perceived reliabale person, aspirants can count on champion

- **Safety**

  Created: 25.05.23 by Olga Siebeck, **Modified:** 21.06.23 by Olga Siebeck

  Comment:
  > Champion radiates safety/ security and calmness; aspirants feel secure in champoion’s presence

- **Setting Boundaries**

  Created: 08.06.23 by Olga Siebeck, **Modified:** 21.06.23 by Olga Siebeck

  Comment:
  > Champion is able to set and maintain boundaries with coworkers and in the professional context; does not let other walk over her/him

- **Social Skills**

  Created: 10.06.23 by Olga Siebeck, **Modified:** 21.06.23 by Olga Siebeck

  Comment:
  > Peer Champion possesses “social skills” - in German: Sozialkompetenz. Refers to an overall competence in handling social situations

- **Structured Approach**

  Created: 26.05.23 by Olga Siebeck, **Modified:** 10.06.23 by Olga Siebeck

  Comment:
  > Champion approaches their work and the way they explain things to aspirants in a structured way

- **Supporting**

  Created: 25.05.23 by Olga Siebeck, **Modified:** 21.06.23 by Olga Siebeck

  Comment:
  > Role aspirants feel supported by Champion

- **Taking on responsibility**

  Created: 25.05.23 by Olga Siebeck, **Modified:** 21.06.23 by Olga Siebeck

  Comment:
  > Peer champion takes on responsibility for new tasks or challenges, often without being asked to do so specifically

- ## Team Leadership

  Created: 25.05.23 by Olga Siebeck, **Modified:** 21.06.23 by Olga Siebeck

  Comment:
  > Champion leads the team in a way that participants perceive as effective, supportive, motivational

- ## Team Member (Shared Group Membership)

  Created: 08.06.23 by Olga Siebeck, **Modified:** 21.06.23 by Olga Siebeck

  Comment:
  > Peer Champion is a (trusted) member of the team;
  > Another widely studied role model characteristic that, we would argue, is related to attainability, is shared group membership. This is in line both with the social identity approach's claim that individuals generally believe that it is easier to become like those who share their social identities (Turner et al., 1994) and with the literature on upward comparison which claims that assimilation to an upward target is facilitated by a shared group membership

- ## Thoroughness

  Created: 28.05.23 by Olga Siebeck, **Modified:** 21.06.23 by Olga Siebeck

  Comment:
  > Champion takes their work seriously: Knows the manual, knows how to operate all the machines, pays attention to details

- ## Transfer of Knowledge

  Created: 08.06.23 by Olga Siebeck, **Modified:** 21.06.23 by Olga Siebeck

  Comment:
  > Champion transfers their knowledge about a certain subject to aspirants or makes it accessible to role aspirants in a way that they can comprehend and apply it

- ## Trustworthiness

  Created: 09.06.23 by Olga Siebeck, **Modified:** 21.06.23 by Olga Siebeck

  Comment:
  > Champion is being perceived as a trustworthy person

Behavioural Model

6 Codes:

- **Mechanisms: Vicarious Learning**

  Created: 25.05.23 by Olga Siebeck, **Modified:** 22.06.23 by Olga Siebeck

  Comment:
  > Vicarious learning is a way of learning that allows individuals to learn from the experience of others.

- **Mediating Variables: Expectancy**

  Created: 25.05.23 by Olga Siebeck, **Modified:** 22.06.23 by Olga Siebeck

  Comment:
  > Expectancy refers to an individual's perceived subjective likelihood of success in a certain task or area, for example, the perceived likelihood of passing a difficult math test (Morgenroth et al., 2015)

- **Outcomes: Skill acquisition**

  Created: 25.05.23 by Olga Siebeck, **Modified:** 22.06.23 by Olga Siebeck

- **Role Model Attributes: Level of Role Model Success**

  Created: 25.05.23 by Olga Siebeck, **Modified:** 22.06.23 by Olga Siebeck

- **Role Model Qualities: Goal Embodiment**

  Created: 25.05.23 by Olga Siebeck, **Modified:** 22.06.23 by Olga Siebeck

  Comment:
  > Goal embodiment refers to the degree to which a role model has successfully reached the role aspirant's goal and is thus closely linked with the capacity to motivate a role aspirant to move toward an already existing goal. (Morgenroth et al., 2015)

- **self-efficacy**

  Created: 25.05.23 by Olga Siebeck, **Modified:** 21.06.23 by Olga Siebeck

  Comment:
  > by learning vicairously from their role models in their function as behavioural models, role aspirants increase their self-efficacy and thus their expectancy beliefs, resulting in higher motivation to pursue the goal in question
  > self-efficacy is an important part of goal- related expectations and, according to Bandura (1977a), one source of self-efficacy is social modeling which leads to vicarious learning. In other words, observing someone successfully engaging in a task will increase one's confidence in being able to successfully complete the task oneself. (Morgenroth et. al, 2015)

Inspiration

6 Codes:

- **Attitude**

  Created: 25.05.23 by Olga Siebeck, **Modified:** 21.06.23 by Olga Siebeck

- **Competence**

  Created: 25.05.23 by Olga Siebeck, **Modified:** 22.06.23 by Olga Siebeck

  Comment:
  > Champion is perceived as doing his/her job well, having specialist knowledge and being able to apply it.
  > Definition Oxford dictionary: “The ability to do something well”
  > While Morgenroth et al. don’t define competence related to a certain goal, but rather related to general skills and intelligence, it will be used to in relation to goal-related competences

- **Mechanisms: Changing perception of external barriers**

  Created: 25.05.23 by Olga Siebeck, **Modified:** 22.06.23 by Olga Siebeck

- **Outomes: Goal Adoption**

  Created: 25.05.23 by Olga Siebeck, **Modified:** 22.06.23 by Olga Siebeck

- **Outomes:Goal Reinforcement**

  Created: 25.05.23 by Olga Siebeck, **Modified:** 22.06.23 by Olga Siebeck

- **Role Model Qualities: Desirability**

  Created: 25.05.23 by Olga Siebeck, **Modified:** 22.06.23 by Olga Siebeck

  Comment:
  > Desirability refers to the degree to which a role aspirant per- ceives a role model in a positive light, and such desirability is likely to make a role aspirant want to be like the role model. Moreover, research has demonstrated that the degree to which a leaders’ own behavior can change followers’ behavior depends on the degree to which they are seen as “worthy role models”—in other words, as desirable
  > (Morgenroth et al., 2015)

Representations of the Possible

5 Codes:

- **Mechanisms: Changing self-stereotypes**

  Created: 25.05.23 by Olga Siebeck, **Modified:** 22.06.23 by Olga Siebeck

- **Mediating Variables: Value**

  Created: 25.05.23 by Olga Siebeck, **Modified:** 22.06.23 by Olga Siebeck

- **Outomes: Motivation**

  Created: 25.05.23 by Olga Siebeck, **Modified:** 22.06.23 by Olga Siebeck

- **Role Model Attributes: Reasons for Role Model Success**

  Created: 25.05.23 by Olga Siebeck, **Modified:** 22.06.23 by Olga Siebeck

  Comment:

  > This code has be assigned as a meta-code to all other codes that described why a role model was perceived as successful

- **Role Model Qualities: Attainability**

  Created: 25.05.23 by Olga Siebeck, **Modified:** 22.06.23 by Olga Siebeck

  Comment:

  > “Moreover, a study by Hoyt and Simon (2011) demonstrates that potential role models that are too successful can be detrimental for role aspirant expectancy. This indicates that the ideal degree of success follows an inverted U-shaped curve: If an individual is not seen as successful enough, they are unlikely embody the role aspirant’s goal in achievement settings. However if the individual is too successful, they may seem unattainable and a contrast effect may occur, leaving the role aspirant in an inferior situation than if they were without this potential role model”
  > “Using the terminology from Weiner’s (1979) theory of attribution, people will be most likely to benefit from a role model’s success if said success seems stable, controllable, and internal.”
