# Supplementary figures and images for: Characteristics of a Successful Nurse Peer Champion in the Implementation of Innovative Digital Technologies in Hospitals: A Qualitative Study
Source: PEC Innov. 2024 Aug 31;5:100339. doi: 10.1016/j.pecinn.2024.100339 (PMC11460499; doi:10.1016/j.pecinn.2024.100339)

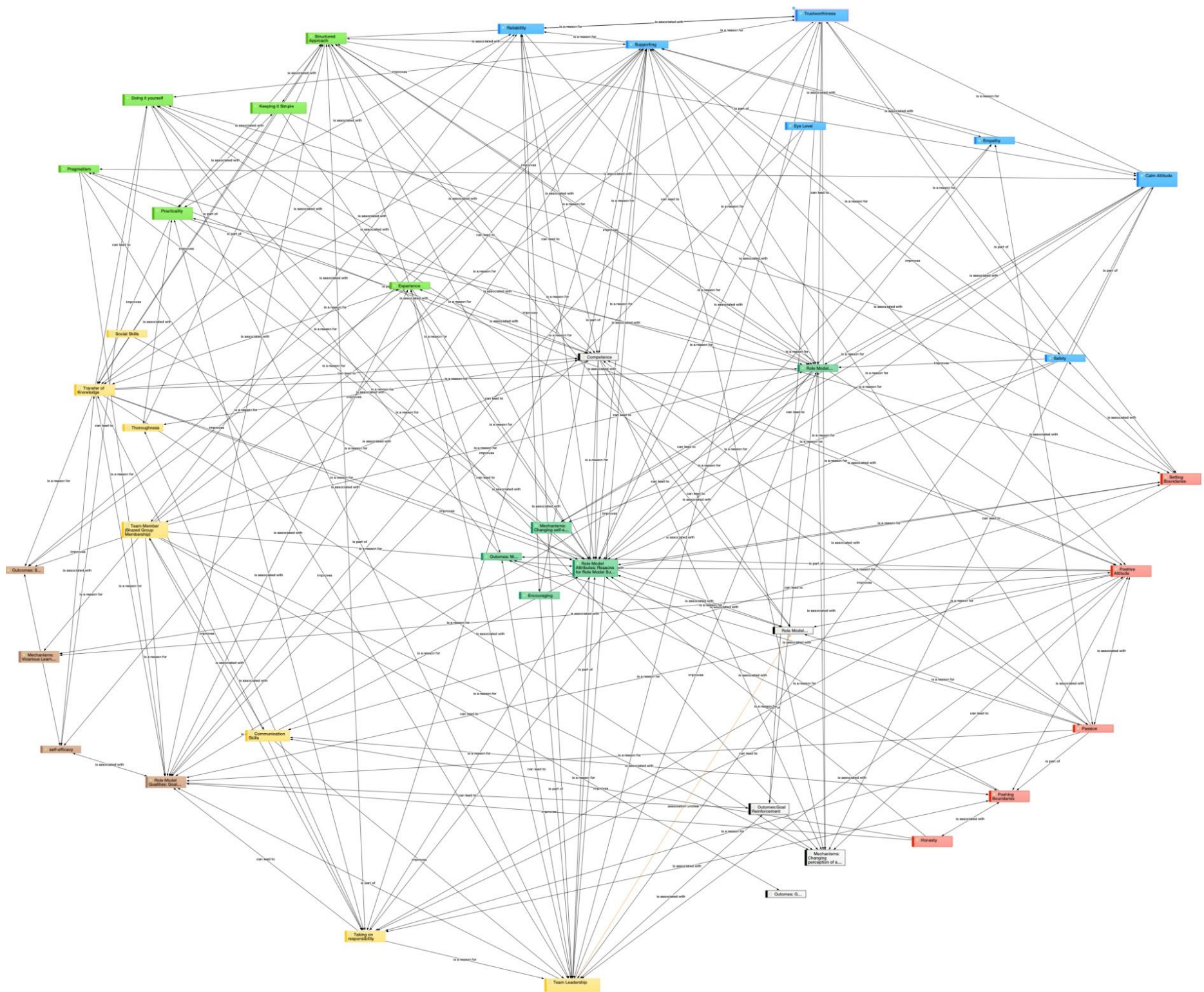

Supplement: Supplementary file 4 — Map of code connections, co-occurences and associations [file mmc4.pdf]
